# Feedback Adaptation to Unpredictable Force Fields in 250 ms

**DOI:** 10.1523/ENEURO.0400-19.2020

**Published:** 2020-04-29

**Authors:** Frédéric Crevecoeur, James Mathew, Marie Bastin, Philippe Lefèvre

**Affiliations:** 1 Institute of Information and Communication Technologies, Electronics and Applied Mathematics (ICTEAM), University of Louvain, Louvain-la-Neuve, 1348, Belgium; 2 Institute of Neuroscience (IoNS), University of Louvain, Brussels, 1200, Belgium; 3 DNAlytics, Louvain-la-Neuve, 1348, Belgium

**Keywords:** adaptive control, feedback control, motor adaptation, reaching

## Abstract

Motor learning and adaptation are important functions of the nervous system. Classical studies have characterized how humans adapt to changes in the environment during tasks such as reaching, and have documented improvements in behavior across movements. However, little is known about how quickly the nervous system adapts to such disturbances. In particular, recent work has suggested that adaptation could be sufficiently fast to alter the control strategies of an ongoing movement. To further address the possibility that learning occurred within a single movement, we designed a series of human reaching experiments to extract from muscles recordings the latency of feedback adaptation. Our results confirmed that participants adapted their feedback responses to unanticipated force fields applied randomly. In addition, our analyses revealed that the feedback response was specifically and finely tuned to the ongoing perturbation not only across trials with the same force field, but also across different kinds of force fields. Finally, changes in muscle activity consistent with feedback adaptation occurred in ∼250 ms following reach onset. The adaptation that we observed across trials presented in a random context was similar to the one observed when the force fields could be anticipated, suggesting that these two adaptive processes may be closely linked to each other. In such case, our measurement of 250 ms may correspond to the latency of motor adaptation in the nervous system.

## Significance Statement

We measure the latency of feedback adaptation in a human reaching experiment by applying force field trials randomly. Despite the fact that these disturbances could not be anticipated, we measured improvement in feedback corrections that paralleled standard adaptation. Correlates in muscle recordings occurred within ∼250 ms following movement onset. Such a short timescale of adaptation suggested that rapid adaptation complements feedback control of an ongoing movement. To further test this hypothesis, we demonstrate that indeed participants are able to adapt their feedback responses to different kinds of force fields and directions applied randomly. These findings support the existence of very rapid, possibly online, adaptation in the nervous system.

## Introduction

Humans and other animals can adapt motor patterns to counter predictable disturbances across a broad range of contexts, including reaching, locomotion, and eye movements (Shadmehr et al., 2010; Wolpert et al., 2011; Roemmich and Bastian, 2018). A central question in movement neuroscience is to identify the time scales at which this process can influence behavior. In the context of reaching movements, standard learning paradigms have focused on trial-by-trial learning, such that changes in behavior were documented by contrasting early and late motor performances, often separated by minutes to hours, or equivalently by hundreds of trials (Lackner and DiZio, 1994; Shadmehr and Mussa-Ivaldi, 1994; Singh and Scott, 2003; Smith et al., 2006; Wagner and Smith, 2008). Thus, a clear benefit of motor adaptation is to improve behavior over these timescales, which is of prime importance for instance when we deal with a new tool or environment. The associated neural mechanism must also be beneficial for adaptation to changes occurring over slower time scales such as development and long-term skill acquisition (Dayan and Cohen, 2011).

Besides the improvement of behavior over medium to long timescales, previous studies also indicated that motor learning could be very fast. The presence of rapid adaptation was previously established by observing after-effects induced by a single movement (Sing et al., 2013). Likewise, unlearning was documented after a single catch trial when a force field was unexpectedly turned off (Thoroughman and Shadmehr, 2000). Other studies showed that the timescale of motor learning could be even faster (Braun et al., 2009; Crevecoeur et al., 2020). In the latter reference, it was documented that healthy volunteers could produce adapted feedback responses to the unanticipated force field perturbations during reaching, and after-effects were evoked within an ongoing sequence of movements in <500 ms when participants were instructed to stop at a via-point.

These latter results contrasted with standard models of sensorimotor learning (Thoroughman and Shadmehr, 2000; Baddeley et al., 2003; Smith et al., 2006; Kording et al., 2007), which included multiple timescales but assumed that each movement was controlled with a fixed representation as a consequence of the assumption that the fastest timescale in these models was longer than the movement time. The expression of after-effects in <500 ms challenged this view, as it reveals that adaptation was potentially fast enough to influence movements of similar or longer duration. Thus, motor adaptation could not only support learning across trials but also complement online feedback control.

Evidence for online adaptation was interpreted in the context of adaptive control (Bitmead et al., 1990): a least-square learning algorithm coupled with a state-feedback controller. This technique is based on standard state-feedback control models that successfully capture humans’ continuous and task-dependent adjustments of voluntary movements (Todorov and Jordan, 2002; Diedrichsen, 2007; Liu and Todorov, 2007), as well as feedback responses to mechanical perturbations (Scott, 2016; Crevecoeur and Kurtzer, 2018). Intuitively, the state feedback controller in the nervous system can be viewed as a parameterized control loop, and the goal of adaptive control is to tune this loop in real time by continuously tracking the model parameters (and errors). This model captured both adjustments of control during un-anticipated perturbations, and the standard single rate trial-by-trial learning observed across a few trials, assuming that changes in anticipation across trials may come from rapid feedback adaptation of the previous one (Crevecoeur et al., 2020).

To gain further insight into the timescales of motor adaptation in the brain, we designed this study to address the following key questions: first, we sought to replicate previous findings of adaptation to unpredictable disturbances, and measure precisely the latency of adaptive changes in control from muscle recordings. Second, we sought to test a surprising prediction of the theoretical framework of adaptive control: if the nervous system tracks model parameters in real time, then, in principle, it should be possible to handle simultaneously force fields not only of different directions (clockwise or counterclockwise) but also of different kinds (i.e., with different force components). First, our results showed that feedback responses to unanticipated perturbations became tuned to the force field within ∼250 ms of movement onset. Second, we found that humans were indeed able to produce adapted and specific feedback responses to different force fields randomly applied as catch trials. Our results confirmed the existence of a very fast adaptation of feedback control during movements and provide an estimate of ∼250 ms for the latency of motor adaptation.

## Materials and Methods

### Experiments

A total of 44 healthy volunteers were involved in this study (19 females, between 22 and 37 years) and provided written informed consent following procedures approved by the local Ethics Committee (UCLouvain, Belgium). Eighteen participants performed the first experiment, another group of 18 participants performed the second experiment, and the rest (*n* = 8) performed the control experiment. The data of the control experiment were published in our previous study and were reused here to underline the similarities between feedback adaptation and standard trial-by-trial learning.

**Figure 1.**
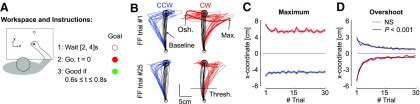
***A***, Illustration of the workspace and task. Participants were instructed to perform forward reaching movements toward a visual target. An open goal target was presented for a random delay uniformly distributed between 2 and 4 s before it was filled in. The cue to reach the target was given by filling in the goal in red. The goal was turned red if the time between the go signal and the stabilization in the target was comprised between 0.6 and 0.8 s. ***B***, Hand paths from the first force field trials (top) and trial #25 selected for illustration (bottom) from each participant (*n* = 18). Counterclockwise and clockwise perturbations are depicted in blue and red, respectively. The black traces illustrate for each panel baseline trials selected randomly (one baseline trial per participant). Osh.: target overshoot; Max.: maximum displacement in the direction of the force field; Thresh.: the positional threshold used to align EMG data. ***C***, Maximum displacement in the direction of the force field. The dashed trace illustrates that the exponential fit did not reveal any significant curvature across force field trials (*p *> 0.05). ***D***, Maximum target overshoot in the direction opposite to the force field. Solid traces revealed strongly significant exponential decay across trials (*p* < 0.001).

In all experiments, participants grasped the handle of a robotic arm (Kinarm, BKIN Technologies), and were instructed to perform visually guided reaching movements toward a virtual target. Each trial ran through as follows. Participants had to wait in the home target (a filled circle with radius 0.6 cm) for a random period uniformly distributed between 2 and 4 s. The goal was also displayed as a circle located 15 cm ahead of the start. After the random period, the cue was delivered to initiate the movement by filling the goal target (Fig. 1*A*). Participants had between 600 and 800 ms (including reaction time) to reach the goal and stabilize in it for at least 1 s. Information about the time window was provided as follows: when participants reached the goal too soon, it turned back to an open circle. When they reached it too late, it remained red. When they reached it within the desired time window, it became green and a score displayed on the screen was incremented. The scores and feedback about timing were provided to encourage consistent movement times, but all trials were included in the dataset. The grand average success rate was 70 ± 12% for experiment 1, and 76 ± 10% for experiment 2. In all cases, the direct vision of the arm and hand was blocked but the cursor aligned to the handle was always visible. These procedures were identical across the three experiments, which only varied by the frequency and nature of mechanical perturbations applied during movements.

#### Experiment 1

This experiment was designed to replicate previous results on the adaptation of feedback responses to unpredictable perturbations (Crevecoeur et al., 2020), and to measure the moment within a trial when the muscle activity started to show feedback tuning corresponding to the force field. Participants performed six blocks of 60 trials, composed of unperturbed trials (baseline) and force field trials. The x and y coordinates corresponded to lateral and forward directions, respectively (Fig. 1*A*). In this experiment, the force field was defined as a lateral force proportional to forward velocity: $f_{x}=\pm l_{O}\dot{y}$, with $l_{O}=\pm 13$ Nsm^−1^ (the subscript O refers to the orthogonal force field). There were five force field trials per block and per direction (counterclockwise and clockwise), which corresponded to a frequency of perturbation trials of 1/6, and a total of 30 force field trials for each perturbation direction. The sequence of trials was randomized within each block, such that the occurrence and direction of the perturbations were unpredictable.

#### Experiment 2

The purpose of this experiment was to test further the hypothesis of online adaptive control by alternating different kinds of force fields, which in theory could be handled by online tracking of model errors (Bitmead et al., 1990). To investigate this, we performed an experiment similar to experiment 1, with the addition of curl force field trials randomly interspersed between unperturbed and orthogonal force field trials. The orthogonal force field was identical to experiment 1. For the curl field, both forward and lateral velocities were mapped onto lateral and forward perturbation forces with opposite signs, respectively: $f_{x}=l_{C}\dot{y}$, and $f_{y}=-l_{C}\dot{x}$ with $l_{C}=\pm 15$ Nsm^−1^ (the subscript C refers to the curl field). There were five perturbation trials per force field (orthogonal and curl) and direction (clockwise and counterclockwise), summing to a total of 20 perturbations per block presented in a random sequence. As in experiment 1, participants performed six blocks of 60 trials, composed of 40 baseline trials and 20 perturbation trials (perturbation frequency: 1/3).

#### Control experiment

In this experiment, we were interested to measure participants’ behavior in a fully predictable context corresponding to a standard adaptation task. Participants performed a series of baseline trials for training, followed by 180 force field trials (orthogonal force field, clockwise (CW) or counterclockwise (CCW) for the entire series), followed by another series of 180 force field trials in the opposite direction for the entire series. The two series were separated by 20 baseline trials to induce washout between the two adaptation phases. We reused previously published data for this experiment and refer to Crevecoeur et al. (2020) for complementary descriptions of the results.

### Data collection and analysis

The two-dimensional coordinate of the cursor aligned to the robotic handle, and the forces at the interface between the participants’ hand and the handle were sampled at 1 kHz, and digitally low-pass filtered with a fourth-order dual-pass Butterworth filter with a cutoff frequency of 50 Hz. Velocity signals were obtained from numerical differentiation of position signals (fourth order, finite difference algorithm). We collected the activities of two of the main muscles recruited when performing lateral corrections against the perturbations used in our experiment: pectoralis major (shoulder flexor) and posterior deltoid (shoulder extensor). Muscle samples were recorded with surface electrodes for experiments 1 and 2 (Bagnoli Desktop System, Delsys). EMG signals were collected at 1 kHz, digitally bandpass filtered (fourth order dual-pass: [10, 400] Hz), and rectified.

Two events were used as timing references. First, reach onset was defined as the moment when the cursor aligned to the handle exited the home target. Second, we used a position threshold located at 1/3 of the distance between the home and goal targets to re-align the EMG traces offline. The crossing of this position threshold approximately coincided with the peak forward velocity, which allowed reducing the trial-to-trial variability in EMG recordings. Similar conclusions were obtained when all analyses were performed based on traces realigned with respect to reach onset.

Exponential fits were used to quantify the presence of learning on several parameters, including the maximum lateral hand displacement, and maximum target overshoot for experiment 1. The quantification of learning from experiment 2 was based on exponential fits of the path length computed as the time integral of hand speed. We fitted the exponential functions to the raw data from each participant as a function of the trial index and assessed whether the 99.9% confidence interval for the parameter responsible for the curvature of the fit included or not the value of 0 (*p *<* *0.001). Variability across participants was illustrated on hand trajectories by calculating the dispersion ellipses based on singular value decomposition of the covariance matrices at different time steps evenly spaced.

We measured both the onset of changes in EMG responsible for changes in behavior across early and late force field trials, as well as the onset of changes in EMG across force fields from experiment 2. To contrast early and late trials, EMG data were averaged for each participant across the first four and last four trials. To contrast the feedback responses to orthogonal and curl fields in experiment 2, EMG data were averaged across the last 15 trials of each kind of force field. EMG averages were then collapsed into a 30-ms-wide (centered) sliding window, and sliding comparisons across time were performed with paired *t* tests. We searched in the time series of *p* values the moment of strongest statistical difference across populations of EMG data (*p *<* *0.005), and then went back in time until the threshold of *p *<* *0.05 was crossed. On the one hand, this test could identify early differences since it included data from –15 to +15 ms relative to the center of the bin, but on the other hand, we kept the threshold of significance instead of attempting to find the true onset of changes in responses that must have occurred a little before. This criterion, along with the fact that the crossing of the threshold of 0.05 was followed by highly significant differences, ensured reliable conclusions. It should be noted that corrections for multiple comparisons do not apply here for two reasons: first, the samples at each time step are involved in only one comparison, and second consecutive samples are not statistically independent. Indeed, if there is a significant difference at a given time step, it is very likely that there is also a significant difference at the next time step because signals do not vary instantaneously. Hence, the risk of false-positive must not be controlled.

An index of motor adaptation was derived based on the relationship between the lateral commanded force and the measured force along the same axis. Similar metrics were used previously (Crevecoeur et al., 2020), and were based on the fact that these correlations were sensitive to learning. A classical approach is based on error clamps or force channels producing virtual walls and allowing an ideal measure of participants’ expectations. In our case, we are interested in online control and force channels are not suitable. The correlations between commanded and measured forces are also impacted by other factors including the robot dynamics (which putatively also influences the force produced against a channel wall), and real-time sampling errors. Besides these errors, the difference between measured and commanded force simply corresponds to lateral acceleration. We documented previously that errors made by ignoring the robot dynamics and by online sampling were on the order of ≤10% (Crevecoeur et al., 2020). Hence, the change in correlation in the range that we observed with similar forward kinematics could be linked to adaptation. It is clear that this metric is close to others that could be captured based on lateral acceleration, velocity or displacement. However, we privileged the correlation as this technique allows extracting a single parameter from continuous curves, by taking their entire time-varying profiles into account.

The data of the control experiment were also used to validate this argument empirically. Specifically, for each trial, we computed a least-square linear regression between the commanded force obtained from velocity signals, and the applied force measured with the force encoders. These correlations were then averaged across perturbation directions for each participant (as they revealed qualitatively similar effects), then across participants for illustration. Surrogate correlations were obtained by calculating linear regressions between the measured force and the commanded force of randomly selected trials with replacement. These surrogate correlations were calculated on 100 randomly picked trials with a replacement for each index and participant.

## Results

### Experiment 1

Our first experiment was designed to replicate previous findings of the adaptation of feedback responses to unpredictable disturbances (Crevecoeur et al., 2020), and measure accurately from EMG data the moment when the perturbation-related activity started to be tuned to the force field. Importantly, a feedback response is expected in all cases (Milner and Franklin, 2005; Wagner and Smith, 2008; Cluff and Scott, 2013). What we searched for was not just a feedback response, but a change in feedback response across early and late force field trials indicating that the response became adapted to the force field. The critical aspect of our experiments with respect to these previous studies, which also documented changes in feedback response, is that in our case, the force field perturbation could never be anticipated.

We measured a clear deviation in the lateral cursor displacement in the direction of the force field as expected since the perturbations could not be anticipated (Fig. 1*B*). Although the maximum hand displacement exhibited a small reduction across the first few trials, the exponential fits of this variable as a function of the trial index did not display any significant curvature (*p *>* *0.05 for both directions). In other words, the parameter in the exponential fit responsible for the curvature of the function was not distinct from 0, reducing to a linear (possibly horizontal) best fit. In contrast, the maximum target overshoot exhibited a clear and highly significant exponential decay across trials (Fig. 1*D*; *p *<* *0.001). Hence, participants initiated force field trials with a controller that would otherwise produce a straight reach path (Fig. 1*B*, black traces), resulting in a clear perturbation-related movement error, but then managed to improve their online correction across perturbation trials.

**Figure 2.**
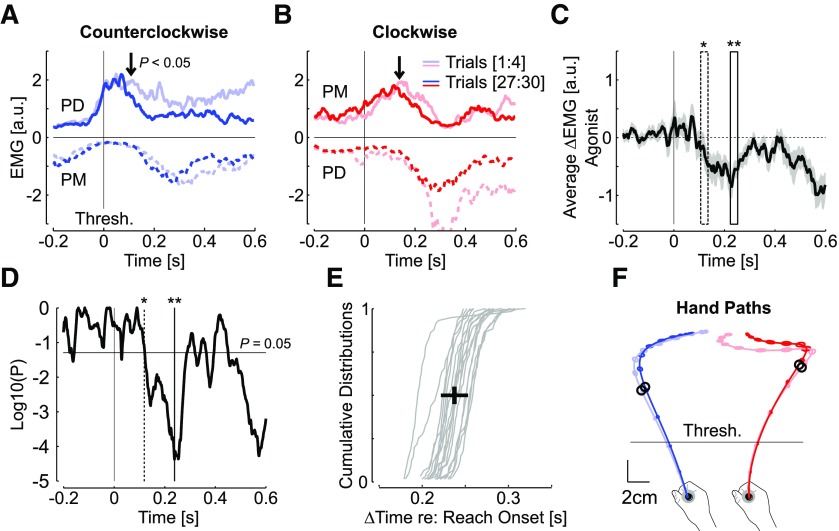
***A***, Activity of posterior deltoid (PD; agonist, solid), and pectoralis major (PM; antagonist, dashed) averaged across the first four (light blue) and last four counterclockwise (dark blue) perturbation trials. The vertical arrows illustrate the moment when a sliding paired comparison of the agonist activity averaged in a 30-m window dropped below *p* < 0.05. Traces were aligned to the position threshold corresponding to one-third of the reach path to reduce variability. ***B***, Same as panel a for clockwise perturbation trials. The position threshold is also represented. ***C***, Grand average of the difference between the agonist activities of the first four and last four force field trials, aligned to the position threshold and averaged across muscles and participants (*n* = 18). The gray area corresponds to the standard error of the mean. The dashed window is the first window that displays a significant difference from sliding paired comparison (*p < 0.05, width = 30 ms). The solid window is the window associated with the minimum *p* value (**p < 10^−4^). ***D***, *p* value of the sliding paired comparison performed on the data from ***C***. All EMG traces were smoothed with a 5-ms sliding window for illustration purposes. ***E***, Cumulative individual distributions of the delay between movement onset, and the moment when the *p* value of panel d dropped below 0.05. This moment corresponds to the time of threshold crossing plus 122 ms. The median delay between movement onset, and this time was 237 ± 15 ms (mean ± SD across participants, *n* = 18). ***F***, Average hand paths and standard dispersion ellipses for the first four and last four trials in each direction, Dispersion ellipses are displayed 50 ms (see Materials and Methods). The black dots represent the moment corresponding to the vertical arrows of ***A***, ***B***.

It was shown previously that the reduction in target overshoot was associated with a reduction in measured force near the end of the movement (Crevecoeur et al., 2020). This result was also replicated below. As a consequence, we expected to measure a reduction in muscle response to the perturbation. To observe this, we averaged EMG data across the first four and last four trials for each perturbation direction. Our rationale was that fewer than four trials would likely be too small a sample, whereas trials performed after five or more force field (FF) trials already exhibited significant adaptation, thereby reducing the size of the effect under investigation. As expected, we found a significant reduction in EMG responses to the perturbations (Fig. 2*A*,*B*). When traces were aligned to the position threshold and averaged across directions (see Materials and Methods), we found that the onset of a drop in the time series of *p* values occurred on average at 122 ms following threshold (center of the 30-ms bin; Fig. 2*C*,*D*). It could be observed that there was no clear difference across the first and last trials before the moment when the *p* value dropped (Fig. 2*C*), which indicated that there was no systematic coactivation of this pair of antagonist muscles on average, and therefore no clear reliance on limb impedance control.

Because there was some variability between reach onset (defined as the moment when the cursor exited the home target) and the moment when participants’ hand crossed the position threshold, we calculated for each subject distribution of elapsed time between reach onset and the moment corresponding to threshold +122 ms. These distributions are reported in Figure 2*E*, and the mean ± SD of medians is shown (black cross): 237 ± 15 ms. For illustration, we reported in Figure 2*F* the mean latency of within-trial changes in feedback response on the average hand path represented for the first and last four trials. The black circle illustrates the moment of a significant reduction in perturbation-related EMG that could be linked to the reduction in target overshoot observed in Figure 1. The analysis of antagonist muscle activity also revealed a decrease in activity in absolute value, but the difference was delayed relative to the onset of change in agonist activity (Fig. 2*A*,*B*, dashed traces). Following the same technique of sliding window, we found that the difference between early and late trials became significant at 400 ± 15 ms (medians across individual distributions, mean ± SD).

**Figure 3.**
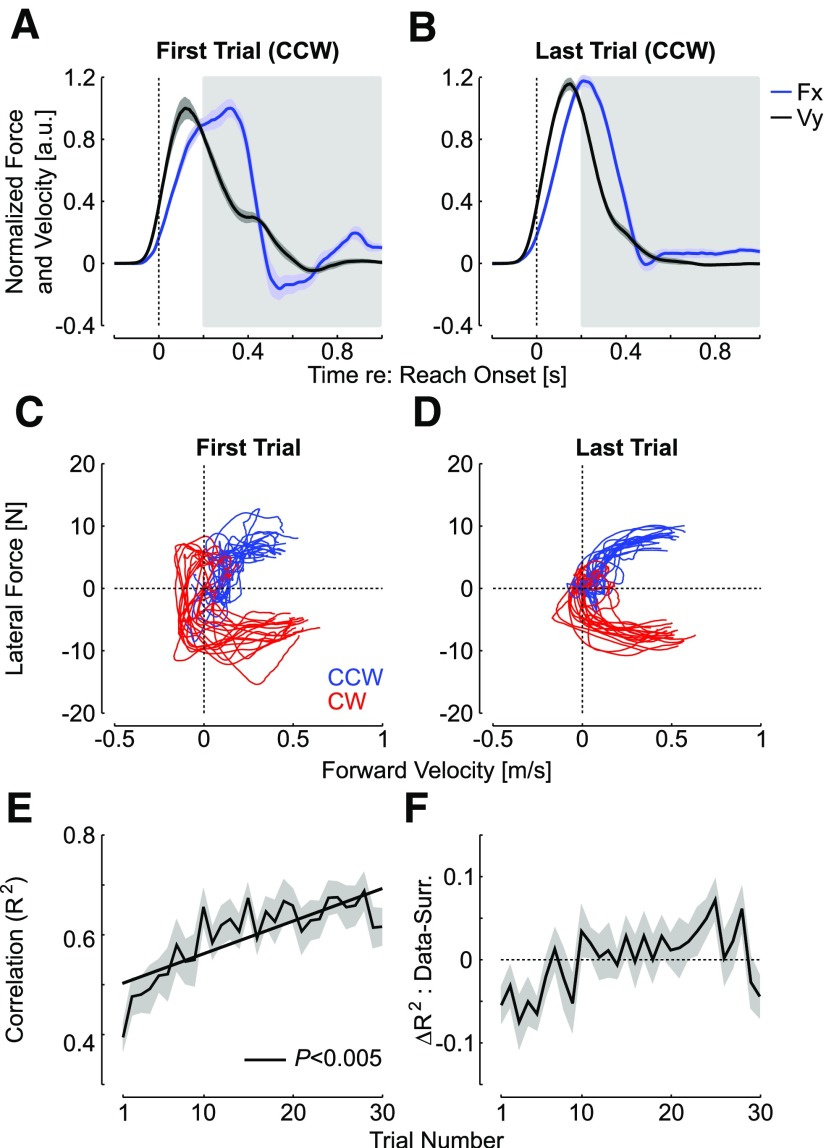
***A***, Forward hand velocity (black) and measured lateral force (blue) normalized to the average maximum calculated on the first trial. Shaded areas represent one standard error across participants (*n* = 18). Panels display data from the trials with counterclockwise force field perturbation. ***B***, Same as panel a for the last trial normalized to the average maximum of the first trial. ***C***, Lateral force as a function of the forward velocity for CW and CCW perturbations (red and blue, respectively). ***D***, Same as ***C*** for the last trial. For panels ***C***, ***D***, there was one trace per participant. Observe that the traces were smoother in the last trials. ***E***, Mean ± SEM of the linear correlation (*R*^2^ statistics) across force field trials from experiment 1 (CCW and CW perturbations averaged). ***F***, Difference between the correlations from experiment 1 as calculated in ***C***, and the correlation between the lateral force of each trial with the forward hand velocity of a randomly picked surrogate trial with replacement. The surrogate correlations were calculated 100 times per trial and participants and averaged across. The shaded area is one SEM.

The reductions in perturbation related response in EMG and in target overshoot were expected if participants learned to handle the force field. To further address whether their online corrections reflected adaptation, we correlated the measured lateral force with the commanded force calculated offline based on forward hand velocities. Average traces were represented in Figure 3*A*,*B* for CCW perturbations (normalized for illustration). Observe that the average correction in the first trial was variable and the traces were irregular (Fig. 3*C*). In contrast, the same data plotted for the last trials appeared more regular (Fig. 3*B*,*D*). Figure 3*C*,*D* shows phase diagrams with measured and commanded forces in the first and last trials for each participant. These traces were taken from ∼200 ms following reach onset to 1000 ms (Fig. 3*A*,*B*, gray rectangles), based on the previous analysis revealing that there was no difference until ∼240 ms following reach onset, and thus no expected improvement in correlation before this time. It can also be observed that the peak terminal force occurring after 400 ms decreased in absolute value, which is consistent with our previous observation (Crevecoeur et al., 2020), and can be linked to the fact that participants were able to reduce the lateral target overshoot when exposed to the force field.

The correlations exhibited highly significant changes across trials. This was first assessed with a repeated-measures (rm)ANOVA on the correlations with the trial indices as the main factor (rmANOVA, *F*_(29,493)_ = 8.7, *p *<* *10^−5^), and a standard least-square linear regression highlighted a clear increase in this variable (Fig. 3*E*; *p *<* *0.005). These correlations were compared with those obtained with randomly picked surrogate profiles to see whether the measured force in each perturbation trial reflected tuning to the ongoing perturbations (see also Materials and Methods; Fig. 3*F*), or whether a non-specific correction pattern was produced, which could correlate as well with randomly picked surrogates as with the experienced perturbation. We found that the true correlations were initially below the surrogate and then became greater than the surrogates. In support of this observation, we found a significant interaction between the the trial index and correlation type (i.e., true vs surrogate, rmANOVA, *F*_(29,493)_ = 1.88, *p *=* *0.004).

This result must be interpreted as follows: when perturbations are associated randomly to the measured forces, it produces surrogate correlations that are initially better than in the data due to more regular profiles taken from late learning, and then it produces correlations that are weaker than the data as early trials with more perturbed profiles are sometimes randomly picked in the surrogates. As a consequence, the change in correlations is not simply due to the fact that the force profiles become more regular, which explains the increase in the surrogate data, but the true correlations are better than those obtained by random associations between commanded and measured forces. In other words, the relationship between the specific perturbation and force profiles from the same trials must be preserved to reproduce the observed correlations. To make sure that this result was not too dependent on some very particular traces that occurred early during learning, we performed the same analyses after removing the first four trials for the surrogate correlations and found the same result (interaction between trial index and true or surrogate types without the first four trials, rmANOVA, *F*_(29,493)_ = 1.9, *p *<* *0.002).

**Figure 4.**
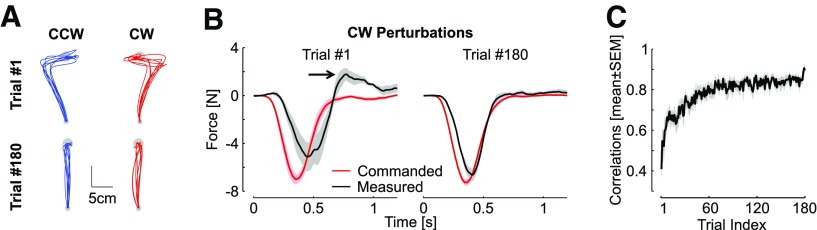
Control experiment, data from Crevecoeur et al. (2020). ***A***, Individual hand traces from the first and last perturbation trials in each direction from the control experiment (one traces per participant, *n* = 8). ***B***, Commanded (black) and measured (red) force profiles for the first (left) and last (right) clockwise perturbations. The arrow highlights the increase in peak terminal force linked with the target overshoot. Observe that the traces become very similar, which increases the temporal correlation between them. ***C***, Correlations between commanded force and measured force as in Figure 3 against trial indices. Correlations were averaged across directions and participants. Displays are mean ± SEM across participants.

We verified with the data from the control experiment that an increase in the same correlations between the measured and commanded forces occurred when the perturbations were fully predictable (Fig. 4). The commanded and measured forces displayed initially variable traces with a terminal increase in interaction force at the handle consistent with the production of a target overshoot (black arrow), followed by more regular and similar profiles. Hence, the key observations were that these correlations represented a sensitive metric of learning, and they increased across trials in the random context of experiment 1 similarly as in the standard context of trial-by-trial adaptation. In all, the data from experiment 1 highlighted that participants were able to adapt their feedback responses to unanticipated force field disturbances within ∼240 ms following reach onset and their force control during unpredictable force field trials paralleled the behavior observed in a standard learning paradigm despite the lack of anticipation.

### Experiment 2

**Figure 5.**
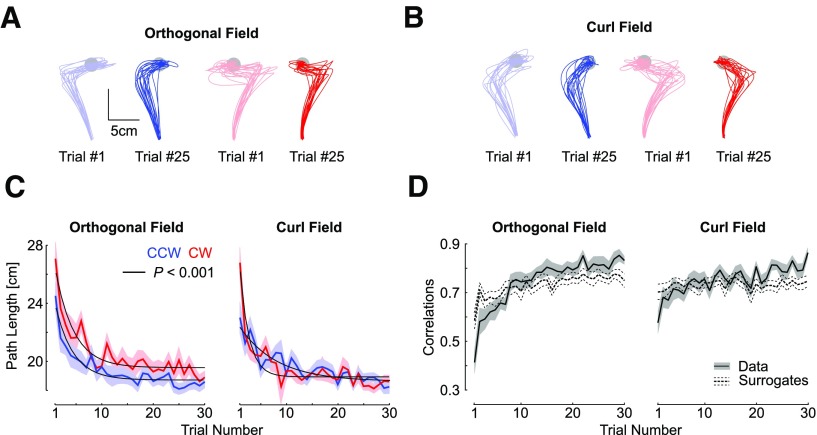
***A***, Hand paths from the first (light colors) and 25th (dark colors) trials with an orthogonal field chosen to illustrate changes in feedback responses. All data were taken from experiment 2, and each trace represents trials taken from each participant (*n* = 18). Blue and red traces represent counter clock-wise and clockwise perturbations. ***B***, Same as panel a for the curl field. ***C***, Path length across force field trials. ***D***, Trial by trial correlations between the lateral commanded force (proportional to forward velocity) and the measured force. Correlations were averaged across counterclockwise and clockwise directions. The shaded areas represent one SEM across participants. The dashed traces (mean ± SEM) are the correlations between the measured lateral force and the commanded force corresponding to the velocity of randomly picked trials with replacement. The procedure was repeated 100 times for each participant, and the results were averaged across CCW and CW perturbations directions.

This experiment was designed to investigate whether participants could learn to adapt their feedback responses when exposed to four different force fields at the same time: either orthogonal or curl force field, in clockwise or counterclockwise directions. For the orthogonal field, we observed the same behavior as in experiment 1: minute changes in the maximum lateral displacement across the first few trials, and highly significant exponential decay of the maximum target overshoot across trials (data not shown). Hand traces during curl field trials were distinct because, unlike the orthogonal field, the anteroposterior component of the force field prevented a systematic target overshoot (Fig. 5*A*,*B*). For this reason, we used the path length to capture adaptation across the two force fields with the same metric. We found a clear reduction in path length for each force field and each perturbation direction (Fig. 5*C*). Exponential fits confirmed a very strong decay across trials (Fig. 5*C*; *p *<* *0.001).

As for experiment 1, we calculated the temporal correlations between the commanded and measured lateral forces averaged across clockwise and counterclockwise directions. For the two kinds of force fields, we found a clear impact of the trial index on the correlations, which confirmed the visible increase shown in Figure 5*D* (rmANOVA, *F*_(29,493)_ > 4, *p *<* *10^−6^). Furthermore, for the two kinds of force fields, we found highly significant interactions between the trial index, and the difference between the true and surrogate correlations (*F*_(29,493)_ > 5, *p *<* *10^−10^). As for experiment 1 we verified that this result was not too dependent on the early phase of learning where force and velocity profiles were more irregular and found also a strong interaction between the trial index and the correlation type after removing the first four trials (*F*_(29,493)_ > 5, *p *<* *10^−10^). Observe also that in this experiment, the correlations in the last trials were significantly stronger than for the surrogate (direct comparisons, one sided paired *t* test: orthogonal field: *t*_(17)_ = 3.7, *p *=* *0.0016; curl field: *t*_(17)_ = 6.4, *p *<* *0.001). This analysis indicated again that true correlations were lower first, then became greater, which supported that online corrections were tuned to the specific force profile experienced during each trial.

**Figure 6.**
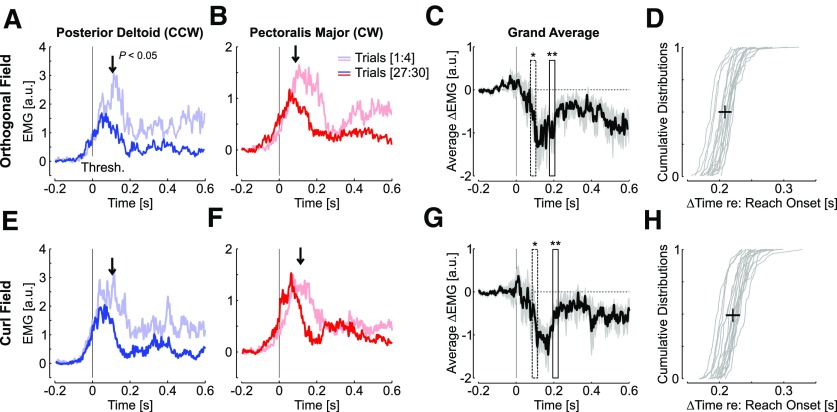
***A–D***, Same as Figure 2 for the orthogonal force field data from experiment 2 (a distinct group of 18 participants). Traces are first and last four trials in the force field for pectoralis major (***A***, blue) and posterior deltoid (***B***, red) aligned to the position threshold. The activity during unperturbed trials was subtracted and the traces were smoothed with a 5-ms moving average for illustration. The vertical arrows show the moment with a sliding paired comparison of activity averaged in 30-ms bins became significant (*p* < 0.05). ***C***, Difference between early and late feedback responses (mean ± SEM) averaged across participants and muscles. The results of the sliding paired comparisons are shown: one star, first 30 ms-bin with *p* < 0.05, two stars: minimum of *p* (*p* < 0.005; see Materials and Methods). ***D***, Individual distributions of time elapsed between reach onset and the center of the first bin with *p* < 0.05. The cross is the median ± SD across participants. ***E–H***, Same as ***A–D*** for the curl force field.

Surface recordings during the orthogonal force fields gave similar results as those reported in Figure 2; we found that the initial responses to the perturbations were similar for pectoralis major and posterior deltoid, until 100 ms following the threshold, where we observed a reduction in activity consistent with the production of an adapted response (Fig. 6*A–C*). As a consequence, the adjustments, in this case, were even observed slightly earlier than during experiment 1. Indeed, the median time elapsed between reach onset, which was defined as the time when they exited the home target, and the reduction in target overshoot was 218 ± 10 ms (Fig. 6*D*). The analysis performed on curl field trials also revealed a significant reduction in perturbation-related activity occurring 104 ms following the threshold, which corresponded to a delay between reach onset and changes in muscle response of 225 ± 11ms (Fig. 6*E–H*).

Again, there was no systematic change in co-contraction, which could have impacted the mechanical impedance of the shoulder joint (Hogan, 1984; Burdet et al., 2001). Indeed, the average traces in Figure 6*C*,*G* represent the average difference in muscle activity aligned to the position threshold for each muscle. The presence of co-contraction would have resulted in an offset observed at the beginning of reach onset. To quantify this, we averaged the activity in each muscle from the first 100 ms of the window presented in Figure 6 (threshold-200 ms until threshold-100 ms), and compared the activity across the first and last four trials. We did not observe any statistical difference (*t*_(17)_ < 1.1, *p *>* *0.3). Besides possible changes in limb impedance (but see Crevecoeur and Scott, 2014), there is a known modulation of baseline activity evoked by unanticipated force field trials identified previously on the same muscles and during a similar task (Franklin et al., 2008; Crevecoeur et al., 2019). However, this effect did not impact the activity before the perturbations systematically, likely due to the fact that perturbation trials were randomly interspersed and the modulation of baseline activity averaged out.

We made similar observations as in experiment 1 regarding the antagonist response: the activity in the antagonist was statistically similar until later in the reach relative to the change in agonist response, for both muscles and for the two force field conditions. We applied again the same sliding analysis to the grand average as suggested in Figure 2*C* but for the antagonist activity and found that the changes relative to reach onsets were at 345 ± 10 ms for the orthogonal force field, and 351 ± 11ms for the curl force field (medians of individual distributions, mean ± SD). These changes in antagonists were also characterized by a reduction in perturbation-related activity.

**Figure 7.**
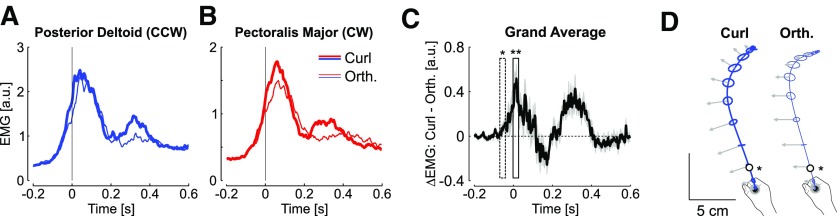
***A***, Average activity of posterior deltoid in curl (thick) and orthogonal (thin) counterclockwise perturbations across the last 15 trials of each type of force field. ***B***, Same as a for pectoralis major from clockwise perturbations. ***C***, Difference between activities recorded during curl and orthogonal force fields averaged across participants and muscles. The onset of significant changes based on a 30-ms-wide sliding window is highlighted with one star (*p* < 0.05), followed by strongly significant differences (two stars, *p* < 0.005). ***D***, Average hand paths during counterclockwise perturbations. Ellipses are two-dimensional standard dispersion across participants every 50 ms, and the impact of the force field is illustrated with the gray arrow (a common scaling for the two force fields was applied for illustration). The open dots are the moment when the activities started to differ across two types of perturbations.

It remained to be elucidated whether the feedback responses to the orthogonal and curl force fields were distinct, or whether participants used a single response pattern undifferentiated across force field disturbance, should the perturbations be sufficiently close to be handled with a single and non-specific response. Our data allowed us to reject this possibility. Indeed, we contrasted the feedback response to curl and orthogonal force fields averaged across the last 15 force field trials for each muscle. We found clear changes in EMG patterns, such that the curl force field evoked a stronger response, and was followed by a second increase in activity near the end of the reach (Fig. 7*A*,*B*). Based on the difference between the activities from curl and orthogonal fields, we found that the modulation of EMG activity was highly significant (Fig. 7*C*). Furthermore, the onset of changes in EMG revealed that the feedback responses were tuned to the force field very early during movement: this time corresponded to ∼55 ms before threshold on average (Fig. 7*D*), which was denoted as a star in the average hand path displayed in Figure 7*D*, black circle. This median of the distributions of elapsed time from reach onset to this time across participants was 65 ± 10 ms (mean ± SD).

It is important to stress that this result was expected and must be used as a control analysis. In fact, the perturbations across orthogonal and curl fields were distinct, thus it is not surprising to observe distinct responses. The goal of this analysis is to verify that the motor system responded to the details of the perturbations across each trial, and as a consequence, there were no default feedback responses triggered in all cases. The link with adaptive control was based on the observation that participants’ behavior had a similar signature as during a trial-by-trial learning scenario (Fig. 4), in conjunction with the fact that there was no anticipation (by design), no systematic co-contraction, and that the feedback responses were adjusted to each perturbation trial (Fig. 7 and surrogate data analyses on Figs. 3, 5).

A consistent observation across Experiments 1 and 2 was a decrease in perturbation-related feedback response, which accounted for the improvement in reach control. We interpret this reduction as an adaptation in the feedback circuits, because of similar changes in correlation as observed in the control experiment with standard trial-by-trial learning. But could there be a “default” downregulation of feedback gains? Our data suggest that it was not the case. We limited this analysis to the orthogonal field for simplicity. Across experiments and perturbation directions, we found no significant variation of forward velocity across trials (rmANOVA, all *F*_(29,493)_ < 1.36, all *p *>* *0.1). Thus, the commanded lateral force was statistically similar. A default downregulation of feedback gains should then be visible in the peak of measured force applied to the handle. We found such a decrease in absolute value in only one case (CCW in experiment 1, *F*_(29,493)_ < 1.74, *p *=* *0.01), and a visual inspection indicated that this happened for the first few trials. In the other cases, we found either no change across trials (*F*_(29,493)_ < 1.2, *p *>* *0.2), or even an increase in the peak of lateral measured force (CW in experiment 2, *F*_(29,493)_ = 2.11, *p *<* *0.001). Thus, there was no default or generic downregulation of feedback gains, instead, it occurred typically later than the peak force during the reach. A precisely timed downregulation with respect to the force field is expected to assume adaptation of feedback motor responses.

To summarize, participants produced feedback responses tailored to the details of the unanticipated perturbations for each kind of force field; these feedback responses improved and exhibited similar traits as those of standard adaptation paradigms, namely the increase in correlation between commanded and measured forces. These changes in behavior were linked to finely tuned EMG activities, which indicated that feedback response was adapted to the force field as early as 250 ms following reach onset.

## Discussion

Current theories of motor learning have postulated that sensory feedback about movement error is mapped to model updates for the next movement. Based on this idea, several seminal studies have characterized learning across movements by means of trial-by-trial learning curves. We recently argued that motor adaptation unfolded over faster time scales, potentially within a single trial, which revealed a novel function of motor adaptation, which is to complement feedback control online (Crevecoeur et al., 2020). To further test this hypothesis, we performed here two experiments with the following aims: (1) to replicate our previous results on improvements in feedback responses to unanticipated force fields; (2) to identify in muscle recordings the latency of adaptive changes in control; and (3) to test whether healthy humans could adapt their feedback response to two different force fields and two different perturbation directions randomly applied across trials. The results confirmed our previous findings and highlighted a modulation of feedback responses within 250 ms of reach onset. Importantly, these feedback responses were also specific to each perturbation profile (curl and orthogonal force fields).

Previous studies investigated learning in random environments and found partial to total anterograde interference between different motor memories dependent on the time between two adaptation sessions (Gandolfo et al., 1996; Shadmehr and Brashers-Krug, 1997; Karniel and Mussa-Ivaldi, 2002; Caithness et al., 2004). To explore the neural basis of acquisition of multiple motor skills, subsequent studies have highlighted that adapting to opposite velocity-dependent force fields was possible if they were associated with different contextual cues, movement representations, initial limb proprioceptive states, or planning conditions (Wada et al., 2003; Addou et al., 2011; Hirashima and Nozaki, 2012; Howard et al., 2012; Green and Labelle, 2015; Sheahan et al., 2016). Our data contrast with this literature at first glance: we claim there was feedback adaptation to opposite velocity-dependent force fields applied randomly without any change in context or representation across trials. We explain this discrepancy by the fact that most studies used indicators such as maximum perpendicular error, based on which we would not conclude for adaptation either (Fig. 1*C*). In contrast, an indication of changes in feedback control occurred later in movement and was observed when we analyzed the details of force, EMG and kinematic signals near the end of movements. Thus, we conclude that there is an adaptation of the feedback controller, which is evoked by the perturbation experienced within the movement, and as a consequence takes some time during the trial before it has a measurable impact on control. Our data suggested that motor execution could form and recall distinct internal representations of dynamics during movement. This interpretation based on adaptive control captures both interference and feedback adaptation: if the nervous system tracks model parameters online, then the alternation of CW and CCW force field trials produces an estimate of the force field that is zero on average. Thus, in this framework, there is no anticipation of the force field type or direction, but the improvements in feedback corrections are mediated by online adaptation. In addition, these individual perturbation responses were not stereotyped. The differences between single-trial adaptive responses when orthogonal and curl field perturbations were randomly intermingled indicated specificity of perturbation response at the single trial level. Interestingly, a field specific adaptation was reported in a random perturbation scenarios were position and velocity-dependent force fields were applied (Joiner et al., 2017). Thus, online responses and single-trial adaptation seem both highly sensitive to the details of the perturbation profiles.

Of course, this adaptation may differ from anticipatory or feed-forward adaptation. In standard learning paradigms, it was postulated that reaching movements consisted of a feed-forward component, which we can define as an open-loop control sequence, along with a feedback component (Kawato, 1999), and that adaptation was associated with changes in the internal forward model (Shadmehr et al., 2010; Franklin and Wolpert, 2011). Several reports have then documented that feed-forward and feedback control systems share their internal representations, based on the observation that changes in feedback corrections correlated with the learning of a force field (Wagner and Smith, 2008; Ahmadi-Pajouh et al., 2012; Cluff and Scott, 2013; Maeda et al., 2018). The adaptation that we highlighted here may be a distinct process because there was no possibility for the participants to change their anticipatory compensation for the force field trials, which were unpredictable by design.

However, the observed feedback adaptation may not be functionally distinguishable from the standard adaptation: it enabled adjustments of motor output suitable to the experienced force field. Although feedback adaptation may be dissociable from feedforward adaptation (Yousif and Diedrichsen, 2012; Kasuga et al., 2015), and may not engage the same circuits, there is no reason a priori to dismiss it as a contributor to motor adaptation in general. A possible link between feed-forward and feedback adaptation is that the online representation which is used to adapt the feedback response in a force field trial may be stored and used as a new prior for the next movement. This hypothesis is motivated by the observation that a single force field trial is sufficient to produce significant after-effects in the next trial (Scheidt et al., 2001; Sing et al., 2013; Crevecoeur et al., 2019; also observed in the present dataset). Considering this, a challenging question arises as to how much feedback adaptation contributes to trial-by-trial learning, and how these two putative processes are implemented in neural circuits.

Our reasoning was based on the theory of adaptive control. The basic premise of this theory is that the controller adjusts the parameters of the state-feedback control loop in real time. In principle, there is no lower bound on the time scale of this mechanism, but the instantaneous learning rate should not be too large to prevent instability (Bitmead et al., 1990). The reduction in target overshoot for the orthogonal field (data from experiment 1) without impacting the maximum lateral hand deviation was previously explained in this context (Crevecoeur et al., 2020). The ability to adapt feedback responses to two different randomly applied force fields was also a prediction of this model: if parameters can be tracked online, it does not matter which force field is applied (curl or orthogonal), since sensory feedback of each specific trial can be used to produce a response adapted to the ongoing perturbation.

Another candidate mechanism to counter unexpected disturbances is the use of co-contraction to modulate the limb’s intrinsic properties (Hogan, 1984; Burdet et al., 2001; Franklin et al., 2008), and the feedback gains in a non-specific way (Crevecoeur et al., 2019; Bian et al., 2020). Such a strategy was previously demonstrated to influence feedback control gains across a few trials, which also limits the extent of lateral displacement following disturbances. However, our current dataset requires another explanation because the perturbations were applied randomly, and there was no co-contraction observed on average. Furthermore, an increase in EMG activity expected if a strategy based on co-contraction was used, whereas we documented a decrease in perturbation-related response across the two experiments (as shown in Figs. 2*A*,*B*, 6*A*,*B*,*E*,*F*). The absence of a systematic increase in baseline coactivation questioned the possibility that a default increase in intrinsic limb impedance or control gains was responsible for the adjustments of control.

Contrary to an increase in limb mechanical impedance, our data showed that the change in feedback correction was instead associated with a reduction in muscle activity, raising the question whether this change was based on knowledge of the force field, or whether it was applied in a non-specific manner. One should recall that the forward velocity was relatively constant by design, and we did not measure any systematic change in interaction force applied to the handle until the peak in this variable. Thus, the reduction in control gain was not a default mechanism either, as it was observed only after some time within the trial. In our view, it is difficult to imagine a precisely timed modulation in control gains unrelated to adaptation (albeit not feedforward adaptation), since the function of this mechanism, that is, to improve control, and one of its behavioral signatures (changes in correlations) were the same across random and predictable contexts.

Online tracking of model parameters is a candidate model to explain the change in control occurring within a movement. Indeed, movements were straight on average and force field trials were often separated by several baseline trials. Thus, participants initiated the reaching movements with a controller corresponding to a baseline trial (i.e., without force field), which would, in this case, produce a straight reach path (Fig. 1, black traces). Then, during perturbations, they changed their control to produce feedback responses that became adapted to the force field. This transition between a baseline controller and a controller adjusted to each force field, along with the observation that each feedback response was better adapted without practicing in a predictable context, constituted strong evidence for adaptive control in the motor system.

How much the controller changed within perturbation trials, or between two trials remains a matter of debate. On the one hand, our previous study provided an upper bound of ∼500 ms within which after-effects could be evoked (Crevecoeur et al., 2020). Our current measurements based on EMG indicated that the change in feedback responses, likely based on the same mechanism, occurred within 250 ms. This time window leaves enough room for adjustments of the controller to each force field within the movement. However, it is clear that changes in movement representation also occurred offline, between two trials or over longer timescales (Krakauer and Shadmehr, 2006; Smith et al., 2006; Kording et al., 2007; Dayan and Cohen, 2011).

Further investigations are required to better characterize the components of adaptive control. For instance, our experiments did not allow teasing apart how much vision and somatosensory feedback contributed to feedback adaptation, as the cursor was visible all the time. However, a strong contribution of proprioception is expected: first, this system could produce detailed responses to the smallest perturbations with long-latency delays (50–60 ms; Crevecoeur et al., 2012), which almost certainly contributed to the early changes in EMG data of experiment 2 evoked by very small differences across orthogonal and curl force fields. Furthermore, previous work highlighted that long-latency feedback engaged motor responses that are well captured in a state-feedback control model (Crevecoeur and Kurtzer, 2018). This rapid state-feedback control loop is supported by a distributed network through primary sensory and motor cortices, pre-motor cortex, parietal regions, and cerebellum (Flament et al., 1984; Omrani et al., 2016). Hence, the fastest adjustments to state feedback control could be achieved by tuning the long-latency feedback loop.

Besides the potential contribution of muscle afferent feedback, the fact that changes in feedback responses were detected within 250 ms leaves enough time to engage task-related feedback responses mediated by touch (Pruszynski et al., 2016; Crevecoeur et al., 2017), and vision, which participates in goal-directed feedback control (Franklin and Wolpert, 2008; Scott, 2016), as well as in rapid changes in navigation strategies (Cross et al., 2019). Characterizing the specific contribution of each sensory system constitutes an exciting challenge for future work.

## Synthesis

Reviewing Editor: Trevor Drew, University of Montreal Decisions are customarily a result of the Reviewing Editor and the peer reviewers coming together and discussing their recommendations until a consensus is reached. When revisions are invited, a fact-based synthesis statement explaining their decision and outlining what is needed to prepare a revision will be listed below. The following reviewer(s) agreed to reveal their identity: Adrian Haith.

Reviewer #1

The authors have addressed a number of concerns. I do think this paper presents an interesting set of experiments and helps to clarify the timescale of these very rapid effects that were demonstrated in the now-published Crevecoeur et al., 2020. However, I still have some outstanding concerns about the paper:

Now that I better understand the analysis based on correlating commanded forces and measured interaction forces, I have some new concerns about this analysis. I do think the improving correlation over time (Figure 3e) is an interesting observation, and likely a good indicator of learning. But I don’t think the interpretation is entirely straightforward, because there is so much contamination due to the dynamics of the interaction between the hand and the robot which I at least find difficult to understand clearly.

I still don’t feel the issue with the correlation between the commanded and measured forces is clearly explained in the methods (see lines 185-191). My understanding is that the robot forces are commanded based on hand velocity. A mismatch in forces would lead to lateral acceleration (of the robot arm, I guess?). Since people move in a straight line after they are adapted, there is little lateral acceleration and the commanded and measured forces should be similar, i.e. high correlation. If this is the case, would lateral displacement/acceleration provide a similar proxy for amount of adaptation? OR does the non-ideal dynamics between hand and robot (i.e. delays, robot inertia, etc) lead to some degree of instability when people are not yet adapted, and this leads to a decorrelation between the commanded force and contact force. If this is the case, to what extent is the “learning” associated with learning the force field imposed by the robot so that it doesn’t become unstable, or learning to handle the unstable interaction between the hand and the robot? (perhaps by globally dialing down their feedback responses?)

Even putting aside what the correlations (or lack of) really represent, I have some concerns about how these are then used:

line 313 - “In support of this observation [that force/velocity correlation is stronger in the real data than in surrogate data], we found a significant interaction between the correlation types (true versus surrogate) and the trial index”. This statistic doesn’t in fact support the claim that the correlation is stronger in the real data than in surrogate data. The interaction could be driven by correlations being worse-than-surrogate early in learning, but similar later in learning. To really establish that correlations are better than surrogate late in learning, it would be necessary to specifically test for a difference in correlation strength between real and surrogate data late in learning. Same concern applies to the same analysis for Experiment 2 (line 354).

Also, depending on what trials’ kinematics are sampled for the surrogate data, the surrogate correlations might end up being weak because of occasional peculiar velocity profiles from early learning trials. This is very different from the authors’ argument that correlations in the surrogate data are weaker than the true data because, in the true data, forces are fine-tuned to trial-to-trial variations in movement velocity, but this is not the case for the surrogate data.

Additional comments:

Abstract: “these two adaptive processes may be imbricated” - I’m not really sure what this sentence means, even after looking up “imbricated” in the dictionary.

299 - “e” in this line should instead be an “f”

326 - “In addition, we found increases in correlations...” Is this sentence here by mistake? It seems to just be repeating what has already been said... (assuming “applied forces” and “measured forces” mean the same thing?)

353 - “we found highly significant interactions between the trial index, and the difference between the true and surrogate correlations” shouldn’t this be an interaction between trial index and data “type” (i.e. true vs surrogate)?

480 - no comma needed here

Reviewer#2

I am satisfied with the revisions made to this manuscript.

There are a few minor typographic errors to correct before publication:

Line 15: “...these two adaptive processes may be imbricated...”. I think the word they want to use is “implicated”, but I’m not sure.

Line 94-96: “...stabilize in it during at least 1s.” Use the word “for”.

Line 216-217: “Fig1c”. Should be “Fig1d”.

Line 299: “e. Difference between...”. I think that should be “f. Difference between...”

## Author Response

We wish to thank the two Reviewers and the Reviewing Editor for their thorough and in-depth assessment of our work. We took good note of the concerns that were raised and we have made serious efforts to clarify our message and modify our manuscript following the Reviewers’ suggestions. Please find below a point-by-point reply with references to the changes that were made in the text.

The authors have addressed a number of concerns. I do think this paper presents an interesting set of experiments and helps to clarify the timescale of these very rapid effects that were demonstrated in the now-published Crevecoeur et al., 2020. However, I still have some outstanding concerns about the paper:

We thank the Reviewer for their positive assessment of this version of the manuscript.

Now that I better understand the analysis based on correlating commanded forces and measured interaction forces, I have some new concerns about this analysis. I do think the improving correlation over time (Figure 3e) is an interesting observation, and likely a good indicator of learning. But I don’t think the interpretation is entirely straightforward, because there is so much contamination due to the dynamics of the interaction between the hand and the robot which I at least find difficult to understand clearly.

We understand that the use of correlations is not as straightforward as the use of measured forces against a channel wall, which cancels motion-related effects. Note however that even in this case the push exerted against the wall reflects the anticipation of both robot dynamics and the force field and one may thus object that there is also contamination in this measure. In our case we could not use a channel because we were interested in online control and thus it was necessary to analyse participants’ behaviour while exposed to the force field.

Our argument was developed in detail in our previous study: first it is clear that participants adapted to the sum of robot and velocity-dependent forces, and the error that we made by ignoring the robot dynamics is <10%. The trials performed in the force field are clearly strongly perturbed relative to the baseline trials, thus the overall force during these trials is clearly dominated by the force field. In addition, the large number of baseline trials suggested that participants were adapted to the null (robot) dynamics. Thus, the change in correlation in the range reported is partially contaminated but this error alone is not sufficient to account for all our results.

I still don’t feel the issue with the correlation between the commanded and measured forces is clearly explained in the methods (see lines 185-191). My understanding is that the robot forces are commanded based on hand velocity. A mismatch in forces would lead to lateral acceleration (of the robot arm, I guess?). Since people move in a straight line after they are adapted, there is little lateral acceleration and the commanded and measured forces should be similar, i.e. high correlation. If this is the case, would lateral displacement/acceleration provide a similar proxy for amount of adaptation? OR does the non-ideal dynamics between hand and robot (i.e. delays, robot inertia, etc) lead to some degree of instability when people are not yet adapted, and this leads to a decorrelation between the commanded force and contact force. If this is the case, to what extent is the “learning” associated with learning the force field imposed by the robot so that it doesn’t become unstable, or learning to handle the unstable interaction between the hand and the robot? (perhaps by globally dialing down their feedback responses?)

Indeed, lateral acceleration could also be used, the main problem was that it is not clear which metric of this variable should be taken: peak lateral acceleration? Absolute value or integral? In fact, the peak occurs early and thus the impact of adaptive feedback control is not apparent. The integral of the lateral acceleration should be zero. The peak terminal acceleration (and force) was used in our previous study as it is linked to the target overshoot that decays exponentially across force field trials. This observation was added to the manuscript as it also characterized the current results (Lines 303-306). In contrast, the correlation seemed better to us as it provided a single number characterizing the similarity between two curves over a time interval. We added this justification to the manuscript (lines 197-200).

Regarding the possibility of instable dynamics induced by the robot, participants performed a large number of trials in the null field corresponding to the robot dynamics and the force field was clearly the main factor responsible for perturbation-related motion during force field trials, thus the force field was the dominant factor. However, it is clear that in general participants had to adapt to the force resultant produced by the interaction of robot dynamics and commanded perturbations.

Even putting aside what the correlations (or lack of) really represent, I have some concerns about how these are then used:

line 313 - “In support of this observation [that force/velocity correlation is stronger in the real data than in surrogate data], we found a significant interaction between the correlation types (true versus surrogate) and the trial index”. This statistic doesn’t in fact support the claim that the correlation is stronger in the real data than in surrogate data. The interaction could be driven by correlations being worse-than-surrogate early in learning, but similar later in learning. To really establish that correlations are better than surrogate late in learning, it would be necessary to specifically test for a difference in correlation strength between real and surrogate data late in learning. Same concern applies to the same analysis for Experiment 2 (line 354). Also, depending on what trials’ kinematics are sampled for the surrogate data, the surrogate correlations might end up being weak because of occasional peculiar velocity profiles from early learning trials. This is very different from the authors’ argument that correlations in the surrogate data are weaker than the true data because, in the true data, forces are fine-tuned to trial-to-trial variations in movement velocity, but this is not the case for the surrogate data.

We are not sure we fully understand this comment: the surrogates were obtained by randomly shuffling trial indices so there was no difference in terms of kinematics variables. The fact that late in learning the correlations are stronger than the surrogate is because there are irregular profiles coming from early in learning used in the surrogates. The conclusion is that changes in the correlation between commanded and measured forces are not accounted for by random association of trials, or that the correlation strength is produced by the relationship between velocity and forces taken from the same trial. One can also deduce that the fact that force profiles become more regular accounted partially for the increase in correlation as in the surrogates, but the true correlation based on the preserve association between variables increased beyond this range.

We have reworked our manuscript to clarify this point. First, we clarified the interpretation of the results based on surrogate correlations (lines 332-340). We then checked whether the correlations in the data were stronger than the surrogates at the end of learning and found it was the case for the second experiment (Lines 386-388). Furthermore, for the two experiments we performed the same analyses after excluding the first four trials to make sure that the results were not overly influenced by the early phases of learning. We found similar results (Lines 341-344 and 383-386). We hope this addresses the Reviewer’s concern and that we did not miss the point. We are willing to perform additional analyses if required.

Additional comments:

Abstract: “these two adaptive processes may be imbricated” - I’m not really sure what this sentence means, even after looking up “imbricated” in the dictionary.

We apologize for the use of Fren-glish and corrected the sentence.

299 - “e” in this line should instead be an “f”

Corrected.

326 - “In addition, we found increases in correlations...” Is this sentence here by mistake? It seems to just be repeating what has already been said... (assuming “applied forces” and “measured forces” mean the same thing?)

Indeed, we have reworked the sentence.

353 - “we found highly significant interactions between the trial index, and the difference between the true and surrogate correlations” shouldn’t this be an interaction between trial index and data “type” (i.e. true vs surrogate)?

Corrected.

480 - no comma needed here

Corrected.

Reviewer#2

I am satisfied with the revisions made to this manuscript.

We thank the Reviewer for their positive assessment of our work. We have corrected the typos and chased for others.

There are a few minor typographic errors to correct before publication:

Line 15: “...these two adaptive processes may be imbricated...”. I think the word they want to use is

"implicated", but I’m not sure.

We have reworked this sentence as follows: “suggesting that these two adaptive processes may be closely linked to each other.”

Line 94-96: “...stabilize in it during at least 1s.” Use the word “for”.

Corrected.

Line 216-217: “Fig1c”. Should be “Fig1d”.

Corrected.

Line 299: “e. Difference between...”. I think that should be “f. Difference between...”

Corrected.
